# ﻿Complete mitochondrial genome of the Galápagos sea lion, *Zalophuswollebaeki* (Carnivora, Otariidae): paratype specimen confirms separate species status

**DOI:** 10.3897/zookeys.1166.103247

**Published:** 2023-06-13

**Authors:** Rita M. Austin, Pia Merete Eriksen, Lutz Bachmann

**Affiliations:** 1 Frontiers in Evolutionary Zoology, Natural History Museum of Oslo, University of Oslo, Oslo, Norway University of Oslo Oslo Norway; 2 Department of Anthropology, National Museum of Natural History, Smithsonian Institution, Washington D.C., USA National Museum of Natural History, Smithsonian Institution Washington D.C. United States of America

**Keywords:** integrative taxonomy, mitogenome, museomics, type sequencing, unique species

## Abstract

The endangered Galápagos sea lion (*Zalophuswollebaeki*) inhabits the Galápagos Islands off the coast of Ecuador. We present a complete mitochondrial genome (16 465 bp) of a female paratype from the collections of the Natural History Museum Oslo, Norway, assembled from next-generation sequencing reads. It contains all canonical protein-coding, rRNA, tRNA genes, and the D-loop region. Sequence similarity is 99.93% to a previously published conspecific mitogenome sequence and 99.37% to the mitogenome sequence of the sister species *Z.californianus*. Sequence similarity of the D-loop region of the *Z.wollebaeki* paratype mitogenome is >99%, while the sequence difference to the *Z.californianus* sequences exceeds 2.5%. The paratype mitogenome sequence supports the taxonomic status of *Z.wollebaeki* as a separate species.

## ﻿Introduction

Intrinsic to the fields of taxonomy and systematics is diagnosability. By convention, the name and description of any new species is unambiguously linked to the original name-bearing type specimen (types). Species identification of organisms is dependent on these specimens’ morphological and molecular attributes. While DNA sequencing and identification is increasingly used in modern biodiversity research, new challenges regarding taxonomic diagnosability have arisen because molecular data is infrequently available for type specimens. DNA sequencing of name-bearing type specimens is, thus, of particular importance for taxonomy as it enables explicit assignment of extant populations to known types (e.g., Renner 2016). The increasing number of reports clarifying evolutionary taxonomic and systematics issues exemplifies the importance and utility of type specimen sequencing (e.g., Kehlmaier et al. 2019; de Abreu-Jr et al. 2020; Zhang et al. 2020).

Here, we present a complete mitochondrial genome of the Galápagos sea lion (*Zalophuswollebaeki* Sivertsen, 1953) female paratype, collected in 1925 by Alf Wollebæk from Floreana Island in the Galápagos Archipelago off the coast of Ecuador. Primarily found in the Galápagos Islands archipelago, *Z.wollebaeki* often congregates in small groups on Isla de la Plata off mainland Ecuador (Melin et al. 2018). Considered Endangered by the International Union for Conservation of Nature (IUCN) (Trillmich 2015), the species has been negatively affected by anthropogenic pressures such as introduced diseases and the increasing frequency of El Niño events linked to climate change (Melin et al. 2018; Krüger et al. 2021; Páez-Rosas et al. 2021). In 2018, a limited population size of 17 000–24 000 individuals was reported after a decline of 23.8% in 2015, a year with an El Niño event (Páez-Rosas et al. 2021). As the number and intensity of El Niño events increases, *Z.wollebaeki* is also facing decreasing pup abundance, further impacting the species’ survivability and conservation status (Páez-Rosas et al. 2021).

The taxonomy of the genus *Zalophus*, including the three species *Z.japonicus* (Peters, 1866) *Z.californianus* (Lesson, 1828, as cited in Ellerman and Morrison-Scott 1966) and *Z.wollebaeki*, has been contentious. *Zalophuswollebaeki* was initially recognized as a new species (Sivertsen 1953), morphologically, but was later reconsidered to be a subspecies of *Z.californianus* (Scheffer 1958). More recently accumulated molecular evidence has suggested that *Z.wollebaeki* is a separate species (Wolf et al. 2007, 2008; Schramm et al. 2009; Berta and Churchill 2012; Krüger et al. 2021). When comparing mitochondrial D-loop and cytochrome B sequences, *Z.californianus* and *Z.wollebaeki* were found to be reciprocally monophyletic, and 25 microsatellite loci further revealed numerous private alleles (Wolf et al. 2007). Although, a recent study (Hassanin et al. 2021) reported an uncorrected pairwise distance between *Z.californianus* and *Z.wollebaeki* mitogenomes of only 0.5%, suggesting that they are, in fact, the same species; despite this low percent difference, they ultimately upheld the separate species taxonomy and instead suggested an adjusted divergence time between the two species to be 0.3–0.2 million years (Hassanin et al. 2021). Originally considered morphologically distinct (Sivertsen 1953), molecular assessment of a *Z.wollebaeki* type specimen can therefore provide a direct link to the initial taxonomic description, while also clarifying evolutionary relationships within *Zalophus*.

## ﻿Material and methods

A female paratype, collected in 1925 by Alf Wollebæk from Floreana Island (Natural History Museum of Oslo, Norway, voucher number NHMO-30317) was used for this study. Interestingly, the given type series collection location differs among the original catalog record (“Chatham/San Cristóbol”), Sivertsen's (1953) holotype description (“Floreana/Sancta Maria”), and the Wiig and Bachmann (2013) publication (“Isla San Cristóbol”) for this specimen. Here, we specify Floreana/Santa Maria Island in corroboration with other specimen collection locations and dates from the same expedition (Wollebæk 1934) that specify “Santa Maria”.

Total genomic DNA was extracted from a left front flipper skin biopsy (257 mg) using the QIAamp DNA Micro Kit (Qiagen, Germany) according to the manufacturer’s instructions. Remaining tissue and DNA extract are stored in the scientific collections of the Natural History Museum, University of Oslo, Norway (voucher number NHMO-DMA-30317/6-D). Extracted DNA (2.4 μg) was submitted for custom sequencing (Illumina NovaSeq 2×150 bp) at the Norwegian Sequencing Centre (https://www.sequencing.uio.no).

The obtained 26 164 466 raw reads (SRA number PRJNA805083) were adapter-trimmed and quality filtered using AdapterRemoval2 (Schubert et al. 2016), with a total of 23 297 925 trimmed and merged reads de novo assembled with SPAdes v. 3.13.1 (Bankevich et al. 2012). An initial mitochondrial sequence assembly using MITObim v. 1.9.1 (Hahn et al. 2013), with a *Z.wollebaeki* D-loop sequence (GenBank accession number AM422173.1) as a bait, yielded a 6042 bp contig. The final mitogenome sequence was obtained by blasting the MITObim sequence against the 257 673 SPAdes-generated scaffolds >500 bp in length. The final mitochondrial genome sequence (GenBank accession number OM636180) was annotated using MITOS2 (Donath et al. 2019) alongside other published Otariidae mitogenomes.

Excluding the D-loop region, the *Z.wollebaeki* paratype mitogenome was aligned to 13 other otariid species [including the previously sequenced *Z.wollebaeki* specimen (Hassanin et al. 2021)] and three phocid outgroup species using MUSCLE on the EMBL-EBI server (Kanz et al. 2005) (https://www.ebi.ac.uk/Tools/msa/muscle/).

The maximum likelihood analysis was run on the ATGC Montpellier Bioinformatics platform (http://www.atgc-montpellier.fr) using PhyML (Guindon et al. 2010) under the GTR model and the Akaike information criterion.

## ﻿Results and discussion

**Figure 1.**
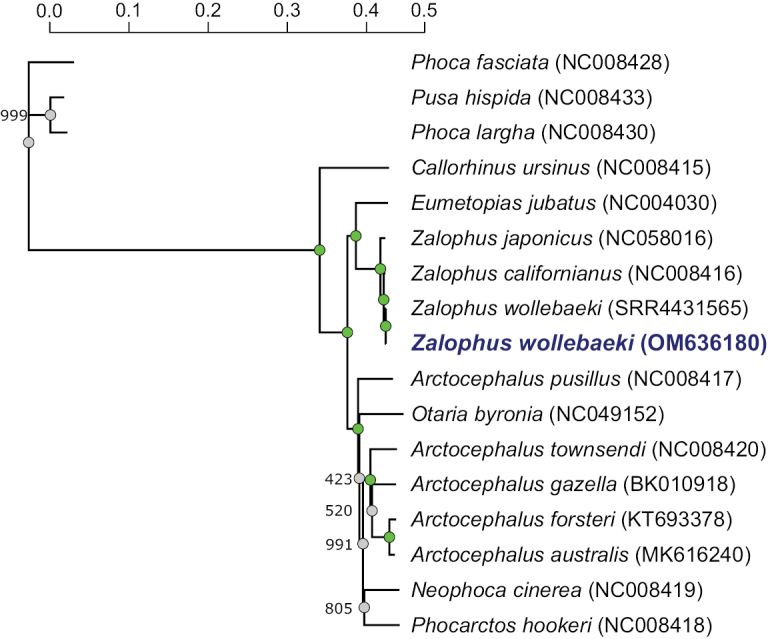
Maximum likelihood tree of several otariid species. Relationships are depicted among the *Z.wollebaeki* paratype mitogenome (bolded and blue), 13 otariid species (incl. the previously described *Z.wollebaeki* mitogenome sequence, GenBank accession number AM422173.1), and three phocid outgroup species. The scale indicates genetic distance. Nodes with 100% bootstrap support (1000 replicates) are depicted in green, with the remaining nodes’ respective bootstrap values indicated to the left.

The mitogenome of the *Z.wollebaeki* paratype was assembled with an average coverage of 36.2X. It is 16 465 bp long and includes all canonical protein-coding sequences, rRNAs, tRNAs, and the D-loop region. Sequence similarity to a previously reported *Z.wollebaeki* (SRR4431565) mitogenome (Hassanin et al. 2021) was 99.93% (12 nucleotide substitutions) and 99.37% to the sister species *Z.californianus*. Accordingly, a maximum likelihood analysis placed the *Z.wollebaeki* paratype sequence in a clade consisting of *Z.wollebaeki*, *Z.californianus*, and *Z.japonicus* (Fig. 1), confirming the authenticity of the paratype sequence.

Comparison with previously published D-loop sequences of *Z.wollebaeki* (Wolf et al. 2007, 2008) revealed a new haplotype that is differentiated from the other 36 haplotypes with one C→T transition (position 15 610). Authenticity of the substitution was confirmed with all 37 reads covering the region sharing the T nucleotide, indicating that erroneous incorporation due to DNA damage can be excluded. The new haplotype is most similar to haplotype Zw_H_10, one of the most common haplotypes reported for *Z.wollebaeki* (Wolf et al. 2008). The sequence similarity of the D-loop region of the *Z.wollebaeki* paratype mitogenome is >99%, whereas sequence differences exceed 2.5% when compared to the 12 *Z.californianus* sequences >600 bp that are listed in GenBank (Wolf et al. 2007). This indicates the *Z.wollebaeki* paratype mitogenome sequence matches well with all nucleotide sequence data reported for more recently collected samples of the species.

The final assembly of the *Z.wollebaeki* paratype mitogenome sequence included 5152 raw reads, which is 0.022% of the adapter-trimmed and quality filtered Illumina readpool. This seemingly low proportion is in the same order of magnitude as observed in other studies. For example, for total DNA extracted from mouse embryonic fibroblasts Quispe-Tintaya et al. (2013) reported a proportion of 0.1% mitochondrial DNA reads, while Anmarkrud and Lifjeld (2017) found 0.03–0.18% mapping mitochondrial DNA reads for historical DNA extracted from museum specimens of extinct birds. The estimated proportion of 0.022% mitochondrial reads is much less than the frequently used rule of thumb stating that mitochondrial DNA represents ~1% of a total DNA extract of mammals. However, the proportion of mitochondrial reads in a next-generation sequencing readpool depends on many parameters such as the extraction protocol, the sequencing methodology, and DNA quality or contamination from other sources. Museum samples are frequently contaminated with external DNA of human origin. Therefore, a control mapping of the adapter-trimmed and quality filtered Illumina readpool to a human mitochondrial genome was conducted. The very low number of 1031 mapping reads indicated that contamination with human DNA is a minor issue, however, this does not mean that the overall sample contamination from external sources is low.

Overall, the complete mitochondrial genome sequence and newly identified haplotype represent valuable genetic references in support of a species distinction between *Z.californianus* and *Z.wollebaeki* using a museum paratype specimen, which may also be constructive for conservation efforts geared toward this charismatic and unique species, and its habitat. With evolutionary relationships within *Zalophus* clarified, genetic assignment of extant populations can now be made more accurately and readily. Furthermore, having an unambiguous connection between the species’ genetic information and the original taxonomic description of *Z.wollebaeki* and paratype specimen, fulfills recent recommendations for incorporating DNA-based species identifications and diagnoses into the various Codes of Nomenclature.

## ﻿Additional information

### Author ORCIDs

Rita M. Austin https://orcid.org/0000-0002-9315-517X

Pia Merete Eriksen https://orcid.org/0000-0003-1832-4794

Lutz Bachmann https://orcid.org/0000-0001-7451-2074

### Data availability

The complete mitochondrial genome and sequencing reads that support this study are openly available on GenBank of NCBI under the accession no. OM636180). The associated BioProject, SRA, and Bio-Sample numbers are PRJNA805083, SRP359162, and SAMN25827133, respectively. The complete mitogenome sequence of *Z.wollebaeki* reported by Hassanin et al. (2021) was obtained from the authors.
